# Conformational transition of FGFR kinase activation revealed by site-specific unnatural amino acid reporter and single molecule FRET

**DOI:** 10.1038/srep39841

**Published:** 2017-01-03

**Authors:** Louis Perdios, Alan R. Lowe, Giorgio Saladino, Tom D. Bunney, Nethaji Thiyagarajan, Yuriy Alexandrov, Christopher Dunsby, Paul M. W. French, Jason W. Chin, Francesco Luigi Gervasio, Edward W. Tate, Matilda Katan

**Affiliations:** 1 Institute of Structural and Molecular Biology, Division of Biosciences, University College London, Gower St, London WC1E 6BT, UK; 2 Department of Chemistry, Imperial College London, South Kensington Campus, Exhibition Road, London SW7 2AZ, UK; 3 London Centre for Nanotechnology, 17-19 Gower St, London, WC1H 0AH, UK; 4 Division of Biosciences, Birkbeck College, Malet St, London, WC1E 7HX, UK; 5 Institute of Structural and Molecular Biology, Department of Chemistry, University College London, Gower St, London WC1E 6BT, UK; 6 Department of Physics, Imperial College London, South Kensington Campus, Exhibition Road, London SW7 2AZ, UK; 7 MRC Laboratory of Molecular Biology, Cambridge Biomedical Campus, Francis Crick Avenue, Cambridge CB2 0QH, UK

## Abstract

Protein kinases share significant structural similarity; however, structural features alone are insufficient to explain their diverse functions. Thus, bridging the gap between static structure and function requires a more detailed understanding of their dynamic properties. For example, kinase activation may occur *via* a switch-like mechanism or by shifting a dynamic equilibrium between inactive and active states. Here, we utilize a combination of FRET and molecular dynamics (MD) simulations to probe the activation mechanism of the kinase domain of Fibroblast Growth Factor Receptor (FGFR). Using genetically-encoded, site-specific incorporation of unnatural amino acids in regions essential for activation, followed by specific labeling with fluorescent moieties, we generated a novel class of FRET-based reporter to monitor conformational differences corresponding to states sampled by non phosphorylated/inactive and phosphorylated/active forms of the kinase. Single molecule FRET analysis *in vitro,* combined with MD simulations, shows that for FGFR kinase, there are populations of inactive and active states separated by a high free energy barrier resulting in switch-like activation. Compared to recent studies, these findings support diversity in features of kinases that impact on their activation mechanisms. The properties of these FRET-based constructs will also allow further studies of kinase dynamics as well as applications *in vivo.*

The central role of protein kinases in physiological processes and their deregulation in a range of diseases have motivated extensive studies resulting in a wealth of knowledge, from biological roles to structural detail at an atomistic level^1^,^2^. Kinases are attractive therapeutic targets and over the past 10 years have become the most important class of drug target in the field of cancer^3^,^4^. However, it is becoming clear that characterization of factors that govern physiological or aberrant activation of protein kinases, or determine drug binding, require further insights into their dynamic features^5^,^6^,^7^,^8^.

Fibroblast growth factor receptors (FGFRs) play a critical role in many physiological processes and have been implicated in pathogenesis of several developmental syndromes and a broad range of human malignancies; a large effort to develop FGFR inhibitors as anticancer treatments is underway^9^,^10^,^11^,^12^. As is generally the case with other protein kinases, structural information alone^13^ is insufficient for understanding their diverse functions or to fully guide drug development. The extent to which protein kinases can be differentiated as dynamic systems is currently unclear, as is the diversity of conformational states they can sample. With respect to activation mechanisms it is possible that inactive kinase conformations could be completely energetically isolated from catalytically active conformations, or that the active state is to some extent energetically accessible, resulting in a preexisting equilibrium of two functionally distinct states.

Experimental approaches to monitor the dynamic properties of protein kinases at high resolution have been based mainly on application of nuclear magnetic resonance (NMR)^14^,^15^, however, this approach has been successfully applied only to a small number of protein kinases^16^,^17^,^18^,^19^,^20^,^21^. Due to the difficulties in obtaining experimental data, molecular dynamics (MD) simulations have been performed more extensively, providing theoretical calculations and working models for dynamic behavior of kinases^21^,^22^,^23^,^24^,^25^,^26^,^27^,^28^,^29^. These studies have also highlighted the need to use a wider range of approaches to illuminate the atomistic dynamics that underpin activation mechanisms or drug binding.

In our studies focusing on FGFR1 kinase domain (KD) we have developed complementary experimental methodology that will facilitate studies of kinase dynamics; we combined site-specific protein labeling with single-molecule Förster resonance energy transfer (smFRET^30^), particularly suitable to detect dynamic behavior within a population^31^,^32^. Furthermore, we have also addressed a major obstacle of non-disruptive labeling of target regions that are key for the activation process, and therefore functionally sensitive, by applying the genetically encoded, site-specific incorporation of unnatural amino acids (UAAs), coupled with rapid and highly specific labeling with a chemical reporter^33^,^34^,^35^,^36^,^37^,^38^,^39^. Importantly, using this novel type of FRET-based construct, combined with MD simulations, we show that FGFR1 KD displays switch-like activation owing to a high free energy barrier between its non-phosphorylated and phosphorylated states, contrasting some recent examples for other kinases supporting preexisting equilibrium^24^,^26^,^40^.

## Results

### FGFR, a model for kinase activation

**Figure 1:**
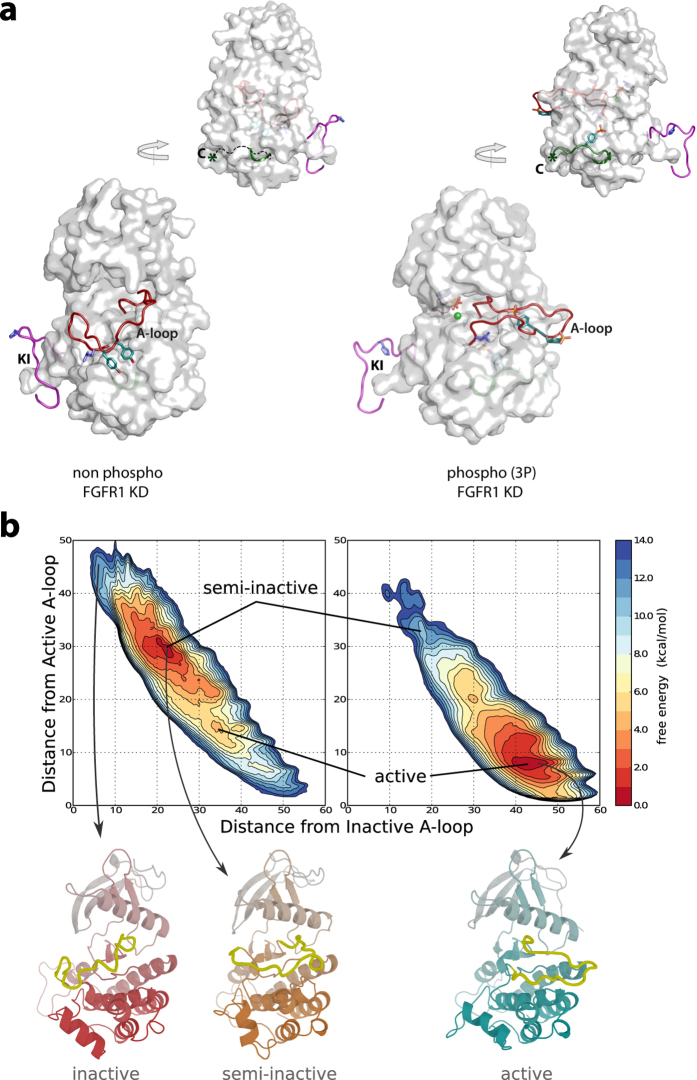
Insights into activation of FGFR1 from crystal structures and MD simulations. (**a**) Crystal structures of non-phosphorylated (pdb: 4UWY) (left) and phosphorylated (pdb: 3GQI) (right) FGFR1 KD are shown as surface representation. The activation loop (A-loop; red) and kinase insert (KI; pink) (bottom views) and the region at the C-terminus (C; green) (top, rotated view) are represented as ribbons. Three tyrosine phosphorylation sites (Y653 and Y654 in the A-loop and Y766 at the C-terminus) are shown in cyan and residues H589 in the KI and L662 in the A-loop are shown in blue. In the non-phosphorylated structure, flexible region at the C-terminus is modeled according to the position in the structure of FGFR1 KD-3P. (**b**) The free energy surfaces of the non-phosphorylated (left) and phosphorylated (right) FGFR1 KD are shown as a function of the distance from the reference inactive A-loop conformation (CV1) and to the distance from the reference inactive A-loop conformation (CV2). The contour lines are drawn every 1 kcal/mol. Selected predicted conformations from the top panels are linked by arrows to their ribbon representations (bottom panels) depicting the following conformations of FGFR1 KD: inactive (corresponding to crystal structure in 1a, left), semi-active (corresponding to the energy minimum) and active (corresponding to crystal structure in 1a, right and the energy minimum).

Kinase domains (KDs) of FGFR1 and FGFR2 have been extensively studied and there are over 70 crystal structures covering inactive states, phosphorylated/active states, complexes with small molecule inhibitors and variants incorporating disease-linked, activating mutations^13^. When a representative, high–resolution structure of inactive, non-liganded FGFR1 KD (pdb: 4UWY) is compared with the active form (FGFR1–3P; pdb: 3GQI) phosphorylated at Y653, Y654 and Y766 (Fig. 1a), repositioning of the activation loop (A-loop) results in a dramatic ~19 Å change in the positions of critical residues Y653 and Y654. The kinase insert region (KI-loop) also appears to adopt a different conformation in the active kinase. In contrast, a defined position of the disordered region at the C-terminus (encompassing Y766) may be determined only after effector binding to pY766 (as in pdb: 3GQI)^41^. Conformational changes within these relatively flexible regions (A-loop and KI-loop) are embedded in an overall relative movement of the entire N-lobe towards the C-lobe in order to facilitate interactions in the catalytic cleft during enzyme catalysis^41^.

Kinetic and energetic properties are very difficult to evaluate from high-resolution crystal structures, even when documented as extensively as in the case of FGFRs. To investigate which conformations are accessible to the respective populations of the KD from non-phosphorylated FGFR1 and FGFR1-3P we calculated the full free energy landscape of FGFR activation from the inactive (A-loop closed) to the active (A-loop open) structure. As the transition takes place on time-scales that are not accessible to conventional molecular dynamics (MD) simulations^42^, we used a combination of metadynamics and parallel tempering (PTMetaD)^43^.

For the non-phosphorylated protein, the main minimum is a semi-inactive structure with a partially distorted activation loop (Fig. 1b, left), thus falling between the two reference conformations, as previously reported for FGFR^27^ and other receptor tyrosine kinases^22^,^25^. In contrast, for the phosphorylated FGFR, the most stable conformation is the active one, with a fully extended activation loop (Fig. 1b, right). The free energy of activation is accordingly 5.0 kcal/mol for non-phosphorylated FGFR and −8.0 kcal/mol for the phosphorylated form suggesting a high degree of separation between the two populations.

### Generation and characterization of FGFR1 KD incorporating genetically encoded unnatural amino acids and tetra-cysteine tag for labeling with fluorogenic reporters

Our approach to experimentally monitor conformational changes of individual proteins combines site-specific incorporation of small fluorescent dyes *in vitro* with smFRET. The sites selected for this type of approach reside in the A-loop, KI-loop and the C-terminus; precise residues within these regions (Fig. 1a) have been selected based on an initial larger screen.

For specific labeling of selected sites within FGFR1 KD (corresponding to H589 and L662), we applied the genetically encoded incorporation of UAAs (bearing a bioorthogonal functional group) in *E. coli* and their subsequent labeling with a chemical reporter *in vitro*. As described previously^35^, the incorporation of UAAs such as BCNK (Nε-(bicyclo[6.1.0]non-4-yn-9-yl-methoxy) carbonyl- L –lysine) determined by reassignment to an amber stop (TAG) codon is based on an engineered pyrrolysyl-tRNA synthetase (PylRS)/tRNA_CUA_^Pyl^ pair from *Methanosarcina barkeri (Mb*). Specifically, a system expressing a variant of *Mb*PylRS (Y271M, L274G and C313A), designated BCN tRNA synthetase (BCNKRS), was utilized on account of its efficient incorporation of BCNK into recombinant proteins^36^. For labeling of the kinase C-terminus, a tetracysteine (TC) motif was introduced that provides a site for specific dye binding that can be performed *in vitro* or in cells^44^.

**Figure 2:**
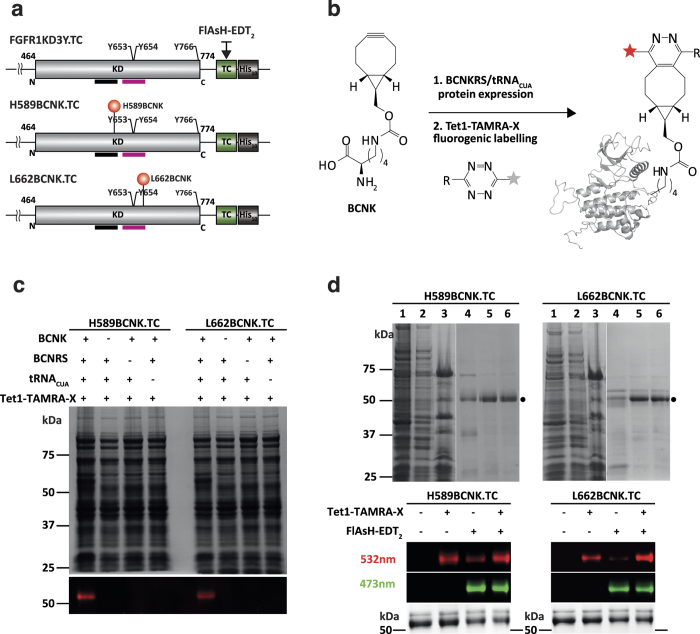
Generation and labeling of FGFR1 KD variants. (**a**) Schematic representation of FGFR1 KD variants (KD; grey), a tetracysteine motif (TC; green) and a C-terminal decahistidine tag (His_10_; black). Three tyrosine phosphorylation sites (Y653, Y654 and Y766) are also indicated. Kinase insert region and activation loop are underlined in black and magenta. The control protein is designated FGFR1KD3Y.TC (top). Two FGFR1 KD variants incorporating replacement of H589 or L662 by BCNK (red spheres) are designated H589BCNK.TC (middle) and L662BCNK.TC (bottom). Incorporation of a TC motif at the C-terminus enables fluorescent labeling with the dye FlAsH-EDT_2_ (black arrow). (**b**) Diagram depicting expression (step 1) and labeling (step 2) of FGFR1 KD via genetically encoded UAA, BCNK. In the first step, BCNKRS/tRNACUAPyl pair is required for incorporation of BCNK to positions corresponding to H689 and L662, directed by reassigned TAG codons. In the second step, a bioorthogonal inverse electron-demand Diels-Alder cycloaddition reaction takes place between the strained alkyne group in BCNK and the tetrazine moiety in Tet1-TAMRA-X. Structures of BCNK and Tet1 are shown while TAMRA-X is represented as a star with increased fluorescence (red) upon protein binding. (**c**) Analysis of incorporation of BCNK into H589BCNK.TC (left) and L662BCNK.TC (right) by TAMRA-X fluorescence. Cell lysates were prepared from conditions lacking one of the indicated components required for BCNK incorporation and incubated in the presence of Tet1-TAMRA-X; following SDS-PAGE, in-gel fluorescence was detected at 532 nm (bottom) and the protein visualized by Coomassie Blue staining (top). (**d**) Purification and labeling of H589BCNK.TC (left) and L662BCNK.TC (right) proteins. Samples obtained at different stages of purification were analyzed by SDS-PAGE; lane 1: *E. coli* pellet before induction, lane 2: *E. coli* pellet after IPTG induction, lane 3: Clarified lysate, lane 4: Immobilized-Metal Affinity Chromatography (IMAC) Ni2+ eluate, lane 5: Steptavidin Trap affinity purification eluate, lane 6: Size Exclusion Chromatography eluate (top panels).Following single or double labeling of proteins and SDS-PAGE, in gel fluorescence was detected at 532 nm and 473 nm and the protein visualized by Coomassie Blue staining (bottom panels).See also Supplementary Figs S1 and S2a.

Constructs used in this study are summarized in Fig. 2a. The control FGFR1 KD protein incorporates 3 tyrosine phosphorylation sites with the C-terminal TC-motif (FGFR1K3Y.TC). Two proteins generated by codon reassignment (H589BCNK.TC and L662BCNK.TC) resulted in either H589BCNK or L662BCNK replacement; both proteins incorporate the TC-motif at the C-terminus (Fig. 2a). To generate a FRET pair, biarsenical dye FlAsH-EDT_2_ (Fluorescein Arsenic Hairpin-binder) was bound to the TC-motif, which forms an N-terminal hairpin structure upon thiol-arsenic ligand exchange reaction and the BCNK residue was specifically labeled with a tetrazine–tetramethylrhodamine (TAMRA) conjugate (Tet1-TAMRA-X) through a Diels–Alder ligation *in vitro* (Fig. 2b, Supplemental Fig. S1). Both FlAsH and TAMRA-X are initially weakly fluorescent, but become strongly fluorescent once attached to the protein.

To verify that BCNK was specifically incorporated into FGFR1 KD variants, we compared *E. coli* lysates prepared from conditions containing all components or lacking either BCNKRS, tRNA_CUA_ or BCNK. Tet-1-TAMRA-X fluorescence, resulting from the binding to FGFR1 KD variants incorporating BCNK, was only observed when all components were present (Fig. 2c). H589BCNK.TC and L662BCNK.TC proteins were subsequently isolated, resulting in a purity >90% (Fig. 2d, top). The time course of protein labeling with FlAsH and TAMRA-X (Supplementary Fig. S2a) was comparable with previous reports for other proteins^36^,^44^. Further single molecule experiments of proteins labeled with FlAsH and TAMRA-X support double labeling of the majority of fluorescent proteins (see next section).

**Figure 3:**
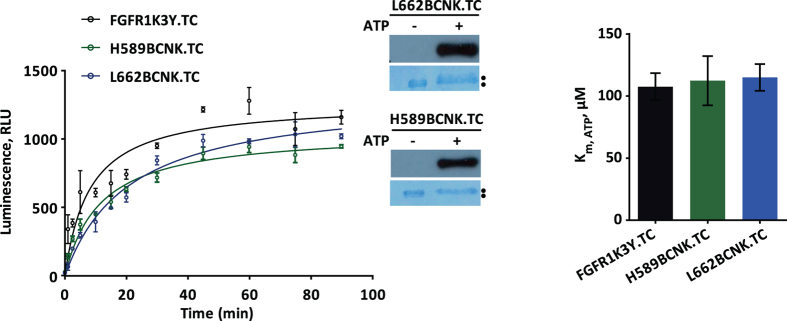
Characterization of FGFR1 KD variants. Kinase activity of FGFR1K3Y.TC (control) and H589BCNK.TC and L662BCNK.TC variants was compared. Time-course of auto-phosphoryaltion was performed using a bioluminescent ADP-Glo kinase assay (left panel). Samples of H589BCNK.TC and L662BCNK.TC before (−ATP) and after 45 min incubation with ATP (+ATP) were subjected to SDS-PAGE and Western blotting using anti-pY653/654 antibody; subsequent staining was with Amido Black (inset). Km values for ATP for FGFR1 KD variants (right panel) were calculated from data obtained in a kinase assay at increasing concentrations of ATP (0–500 μM) using poly(E_4_Y_1_) peptide as a substrate. See also Supplementary Fig. S2b.

To test the functionality of H589BCNK.TC and L662BCNK.TC proteins, kinase activity was compared to FGFR1 KD control (Fig. 3). The ability of H589BCNK.TC and L662BCNK.TC to auto-phosphorylate was retained with only small reduction (Fig. 3, left panel) and the Km values for ATP were similar (Fig. 3, right panel). All proteins reached saturating levels of auto phosphorylation within 60 min accompanied by a shift on SDS-PAGE (Fig. 3, left panel, inset). Our previous characterization of FGFR1 KD-3Y has demonstrated that the key Y residues became phosphorylated under these conditions and result in an increase in kinase activity^27^,^45^. Subsequent binding of FlAsH and TAMRA-X to H589BCNK.TC and L662BCNK.TC had minimal effect on kinase activity (the values were within 20% of the unlabeled protein). Furthermore, when phosphorylated, the activity of labeled H589BCNK.TC and L662BCNK.TC proteins increased from 0.66 ± 0.04 to 2.19 ± 0.36 and from 0.68 ± 0.03 to 2.42 ± 0.24, respectively; this is comparable to the increase from 0.82 ± 0.03 to 2.74 ± 0.31 for unlabeled WT FGFR1 KD (measured as μM of ADP in ADP-Glo™Kinase Assay), demonstrating activation of the labeled proteins due to correct phosphorylation also confirmed using mass spectrometry (Supplementary Fig. S2b).

### Conditions and parameters for smFRET

**Figure 4:**
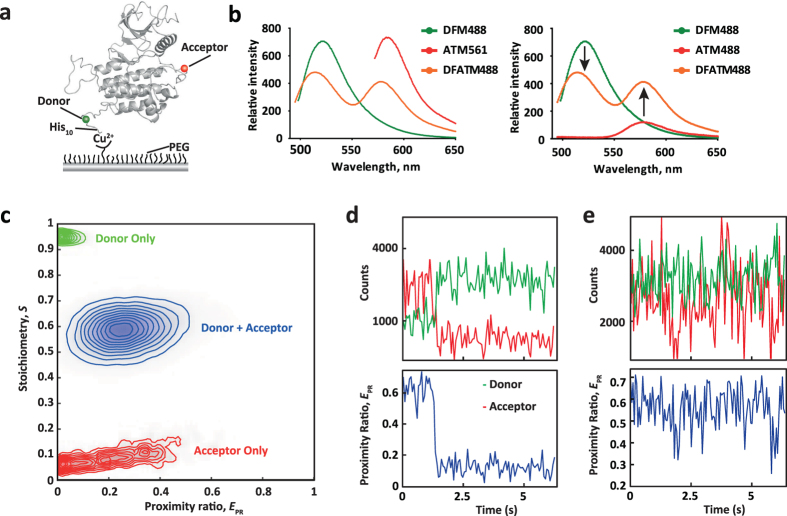
Surface immobilization strategy and correction factor retrieval for smFRET. (**a**) Schematic showing the immobilisation of His-tagged protein to the surface coverslip for smFRET-TIRF experiments. The immobilized protein maintains its native conformation and activity as a result of the controlled orientation and inertness of the chemical surrounding provide by the Cu2+/PEG surface. (**b**) Solution measurements of emission spectra of L662BCNK.TC labelled with either donor FlAsH-EDT2 (DFM) or acceptor Tet1-TAMRA-X (AT) or both dyes (DFMAT) at the fixed excitation wavelength: 488 nm for DFM and DFAMTM and 561 nm for ATM (left). Emission spectra of the same samples, all at the fixed excitation wavelength of 488 nm are also shown (right) clearly demonstrating energy transfer from the donor to the acceptor (arrows). (**c**) Differentiation of donor-only, acceptor-only and donor-acceptor subpopulations by Alternating Laser EXitation (ALEX). A 2D-histogram/contour plot of the uncorrected stoichiometry (S) values versus the apparent FRET efficiency, known as proximity ratio (E_PR_), shows the position of each subpopulation (donor only, green; acceptor only, red; donor + acceptor, blue) determining the contributions of crosstalk of the donor emission into the acceptor detection channel and the direct excitation of the acceptor with the donor excitation laser. FRET efficiency (E_PR_) corrections, including accounting for background photons, are further discussed in Supplementary Material. (**d**) An example of a single Tet1-TAMRA-X acceptor dye photobleaching step, with corresponding increase in donor FlAsH-EDT2 emission. The photobleaching step manifests a drop in the calculated FRET efficiency (E_PR_). (**e**) An example of a corrected fluorescence intensity time trace of donor and acceptor (upper trace, donor FlAsH-EDT2 in green, acceptor Tet1-TAMRA-X in red) from which E_PR_ (lower trace, blue) is calculated. See also Supplementary Figs S3 and S4.

Before protein immobilization for smFRET (Fig. 4a), suitable photophysical properties of the donor-acceptor pair were confirmed for FGFR1 KD proteins in solution (Fig. 4b). The donor/FlAsH-EDT_2_ attached to the TC motif and acceptor/Tet1-TAMRA-X attached to BCNK in FGFR1 KD variants were subjected to excitation and emission scans of the singly and doubly labelled protein (illustrated for L662BCNK.TC in Fig. 4b). The data demonstrate distinct profiles (left panel) and FRET between the two fluorophores at a fixed excitation of 488 nm (right panel).

To monitor conformational transitions and states of FGFR1 KD by smFRET we applied “objective-type” total internal reflection fluorescence (TIRF) microscopy (Supplementary Material, Supplementary Figs S3 and S4), a similar set up as used in previous studies^46^. smFRET-TIRF required that samples prepared in solution are subjected to immobilisation, in this case of histidine-tagged FGFR1 KD proteins to polyethylene glycol (PEG) surfaces using chelated Cu^2+^ groups (Fig. 4a). Also, an oxygen scavenger system with triplet state quencher was used to suppress photo blinking and enhance the photostability of the dyes by reducing photobleaching.

We used alternating-laser excitation (ALEX^47^) of the donor and acceptor dyes to differentiate the observed FRET species in accordance with the number and type of fluorophores present^48^,^49^. To calibrate the measurements, correction factors relating to spectral cross-talk (where the donor fluorescence is visible on the acceptor emission camera) and cross-activation (where the donor excitation laser directly excites the acceptor dye) were determined. These correction factors, in addition to local background subtraction are required for determining intramolecular proximity changes (see Supplementary Material). Single and double labelled L662BCNK.TC proteins were used to determine the correction factors for cross-talk and cross-activation, enabling subsequent calculation of the corrected proximity ratio (E_PR_) and the corrected stoichiometry (S) (Supplementary Material). Singly labelled species showed corresponding stoichiometry centered at about 0.95 for FlAsH only and about 0.05 for TAMRA-X only indicating the presence of single dyes. Double labelled protein showed an intermediate factor of about 0.57, suggesting approximately 1:1 FlAsH/TAMRA-X labelling ratio (Fig. 4c). Cross-correlation analysis of single molecule time traces of donor and acceptor, from which E_PR_ is calculated, show anti-correlated behaviour and demonstrate that intensity fluctuations result from energy transfer (Fig. 4d,e).

### Analysis of inactive and active forms of FGFR1 KD

**Figure 5:**
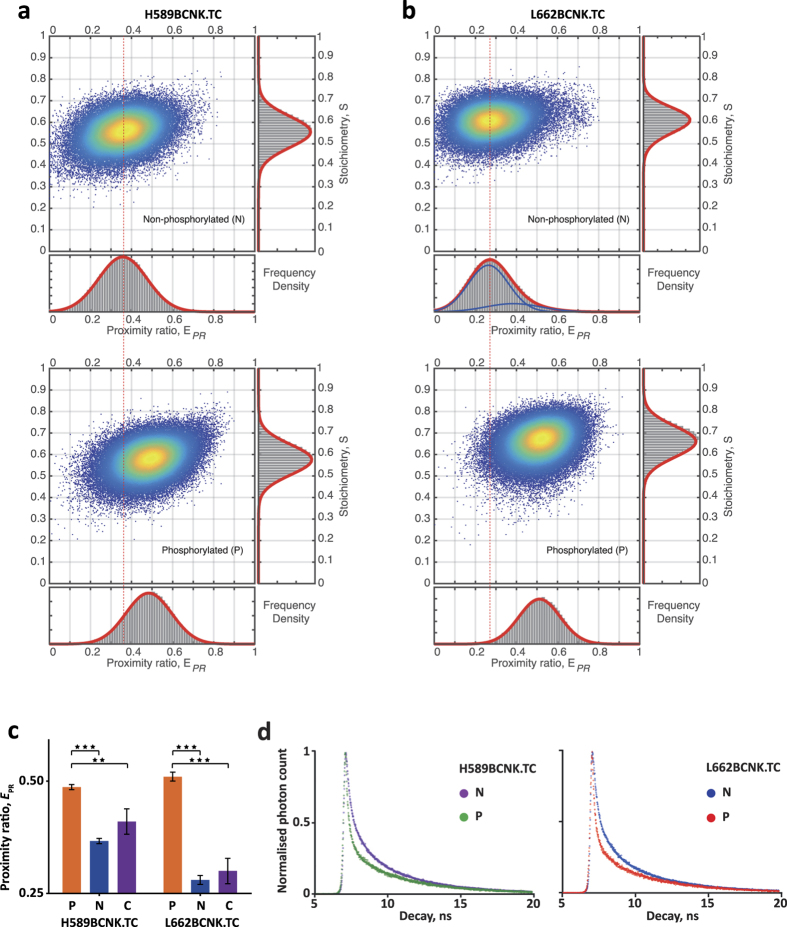
Comparison of labeled, non-phosphorylated and phosphorylated states of FGFR1 KD by smFRET. (**a,b**) *E*_PR_-*S* 2D histogram plots with projections (bottom: *E*_PR_; right: *S*) of double-labeled H589BCNK.TC (**a**) and L662BCNK.TC (**b**) in non-phosphorylated (N) and phosphorylated (P) states for comparison. The 1D-histogram subplots (grey bars) are compared to the Gaussian distribution fits (red lines, subpopulations in blue). The *E*_PR_ 1D histogram subplots show a unimodal distribution of events (counts) in all measured samples except for L662BCNK.TC in the non-phosphorylated form, which is described by a Gaussian Mixture with two components (0.8 and 0.2 respectively) shown in blue. (**c**) *E*_PR_ mean from 3 independent experiments under phosphorylating (P, orange), non-phosphorylating (N, blue) or control, ADP-supplemented (C, purple) conditions. Error bars indicate the standard deviations (SDs). Statistical significance was assessed using one-way ANOVA with Bonferroni multiple comparison test (**0.001 < *P* < *0.01*, ***0.0001 < *P* < *0.001*). (**d**) TR-FRET readout of FlAsH-EDT_2_ and TAMRA-X labeled H589BCNK.TC (left) and L662BCNK.TC (right), comparing non-phosphorylated (N) and phosphorylated (P) samples. The fluorescent lifetime decay of the FRET donor is determined by fitting into a stretched exponential decay model with the measured instrument response function (IRF). See also Supplementary Fig. S5.

Prior to immobilization for smFRET analysis, FGFR1 KD variants were either phosphorylated in the presence of ATP or prepared under control conditions. A few hundred individual fluorescent intensity traces (typically > 1000 counts, ranging between 236 to 406 traces) were analyzed. Representative E_PR_-S two-dimensional histograms of FlAsH and TAMRA-X labeled H589BCNK.TC and L662BCNK.TC proteins are shown in Fig. 5. The population of non-phosphorylated H589BCNK.TC kinase exhibits a peak at E_PR_ 0.38 and a unimodal distribution while the phosphorylated form exhibited a peak shifted to *E*_PR_ 0.49 with a slightly broader unimodal distribution (Fig. 5a). FRET fluctuations for both proteins likely occur on a very fast timescale (similar to, or faster than the duty cycle of ALEX using our EMCCD acquisition rate), and we do not observe protracted dwells in alternative states, as characterised by the unimodal *E*_PR_ distributions. We note a small upward shift in the S peak between non-phosphorylated and phosphorylated forms of both proteins (Fig. 5a,b), suggesting a minor change in quantum yield of one or other of the fluorescent dyes, consistent with a subtle changing of their chemical environment^47^ upon activation of the kinase.

Comparison of non-phosphorylated and phosphorylated forms of L662BCNK.TC kinase revealed an even bigger shift of *E*_PR_ peaks from 0.28 to 0.52 with a similar peak count and a similar unimodal population distribution under phosphorylated conditions (Fig. 5b). Interestingly, under non-phosphorylated conditions, the *E*_PR_ distribution is skewed, suggesting a mixture of two populations, heavily weighted toward a low FRET species (80%) with the remainder (20%) as a higher FRET species, perhaps resembling the active form.

The mean *E*_PR_ from three independent experiments is shown in Fig. 5c, showing a statistically significant change in *E*_PR_ between unphosphorylated and phosphorylated forms for both variants of the protein (*P* < *0.001*). All experiments included an additional control in which protein samples were supplemented with adenosine diphosphate (ADP), instead of ATP, that would occupy the same binding pocket but would not result in phosphorylation. For both variants, the ADP control showed a comparable profile to that of non-phosphorylated forms.

Shift of E_PR_ peaks towards higher values (Fig. 5) implies closer spatial proximity of fluorescent dyes and is likely to reflect a combination of a more compact structure of phosphorylated kinase and specific repositioning of the loop regions. Because FGFR1 KD incorporating all elements required for protein labeling is not structurally defined, our model consistent with smFRET data for the two variants, depicts the possible arrangements of relative distances (Supplementary Fig. S5).

In addition to smFRET we also performed time-resolved fluorescence energy transfer (TR-FRET) studies in solution. Despite some differences in the experimental setup and conditions (described in Supplementary Material), results from TR-FRET using H589BCNK.TC and L662BCNK.TC proteins labeled with the same donor-acceptor pair showed qualitatively similar changes towards closer proximity of the two fluorophores upon phosphorylation, indicated by a more rapid donor fluorescence decay (Fig. 5d). These TR-FRET observations are consistent with the data obtained by smFRET (Fig. 5a–c), demonstrating that both measurements can distinguish and therefore report non-phospho/inactive and phospho/active forms of FGFR1 KD.

## Discussion

Despite extensive studies of protein kinases, insights into their dynamic properties that can provide a bridge between static structure and function remain limited. Progress in this area requires development of new tools including precise FRET-based reporters of dynamic states and conformational transitions and their further analysis using methods such as smFRET, that are capable of capturing large-scale conformational movements. We here describe such new tools developed for one of the key signaling protein kinases, FGFR.

We generated a novel class of FRET constructs that directly report differences corresponding to states sampled by an inactive or an active signaling kinase. The conformational transitions and dynamic range can be measured in solution or using immobilized proteins under physiologically relevant conditions *in vitro* (Fig. 5) and, in future applications, within a cellular environment. These properties have been achieved by specific incorporation of an UAA into key functional regions (linked to activation) of the kinase domain without diminishing its activity and subsequent labeling together with a TC-label to generate a FRET pair (Fig. 2). The labeling protocol for this genetically encoded UAA, BNCK, has been initially applied to model proteins^36^ and now there are several examples of UAA combined with smFRET to study functionally important proteins (*e. g.* p97^37^). Here, we demonstrate that despite a comparatively low yield of FGFR1 KD variants, we were able to purify and characterize these proteins for smFRET measurements (Figs 2 and 3). Methodologically, these FRET constructs can complement NMR studies of protein dynamics *in vitro*^15^,^50^ by accessing regions not visible by NMR, such as highly mobile segments of the A-loop, and bypass limitations of molecular weight and requirement for large protein quantities. Compared to current cellular reporters incorporating GFP derivatives^51^, the future application of these probes could generate direct and localized readouts; they could also provide a basis for a more general strategy covering a wider range of signaling kinases by eliminating interference from bulky (genetically encoded) fluorophores.

Furthermore, through a combination of both MD simulations and smFRET experiments, our data suggest a high degree of conformational separation between the non-phosphorylated and phosphorylated populations of FGFR1 KD. The change in the FRET distances for two FRET-constructs, characterized by a single peak and unimodal distribution for each state (Fig. 5a and b), is consistent with a high energy barrier between a semi-inactive folded structure, the most stable for non-phosphorylated FGFR, and a fully active one that is the most stable conformation sampled by phosphorylated FGFR KD (Fig. 1b). Our findings are also consistent with the presence of a “molecular brake” in an inactive state of FGFR KD and similar kinases^52^, a feature near the hinge region that could restrain two kinase lobes and also suppress the catalytically significant DFG flip in the A-loop by imposing a particularly high free-energy barrier^20^.

The data for FGFR1 KD (Figs 1b and 5a–c) are in sharp contrast with recent observations obtained for a metabolic phosphoglycerate kinase (PGK)^40^. MD simulations and smFRET show equilibrium between two populations (“expanded” and “compact”) for ligand-free PGK; substrate binding only slightly shifts the equilibrium toward the compact state. These data show that PGK is intrinsically a highly dynamic system sampling a wealth of conformations and that catalytically relevant conformations are, to a large extent, already occurring in ligand-free PGK^40^. MD simulations have also been performed for several signaling protein kinases including EGFR, Abl, Src and B-Raf^22^,^23^,^24^,^25^,^26^,^28^. The EGFR KD, similarly to FGFR KD, may not be able to access active conformations to a significant degree unless perturbed by activating mutations^22^,^23^,^25^. Studies of Abl kinase, where the SH2 domain has a role in auto-inhibition and activation^53^,^54^, provide a contrasting example. Recent MD simulations applied to Abl KD in isolation predict an equilibrium between two similarly populated states where the activating role of the Abl SH2 domain have been associated with a shift in the equilibrium and lower free energy of the active state^24^. Similarly, B-Raf may also be an example of a “preexisting equilibrium”^26^. Therefore, protein kinases in their non-phosphorylated state or in the absence of regulatory protein-protein interactions, are likely to differ in the range of conformational states they can sample and sizes of the corresponding populations. Further theoretical and, in particular, experimental approaches such as those applied here to FGFR KD are needed to extend these initial studies to a wider range of signaling kinases and application of further dynamic studies to fully determine implications of their dynamic properties for regulation, subversion in disease and new strategies for treatment.

## Methods

### Protein expression and purification

To express FGFR1 KD with the incorporated unnatural amino acid BCNK, we transformed *E. coli* BL21 (DE3) cells with p*BKBCNRS* plasmid (encodes *Mb*BCNRS) and one of *p*2xStrpII.SUMO*.*FGFR1KD3YTAG*.TC.His_10_. *PylT* plasmids (encode *Mb*tRNA_CUA_^Pyl^ and a multi-tagged variant of FGFR1 KD3Y with an amber TAG codon introduced at a position of H589 or L662 codon). Cells were grown at 37 °C in terrific broth (TB) containing carbenicillin and spectinomycin. At a cell density (OD_600_) of 0.4 to 0.5, BCNK (2 mM) was added to the culture and at OD_600_ of 1.0 to 1.2, cultures were cooled to 15 °C and expression induced with IPTG (400 μM). Following cell lysis, FGFR1 KD variants were purified using HisTrap FF, StrepTactin Sepharose HP, and a Superdex 200 10/300 GL column. See Supplementary Material for a detailed description.

### Protein labeling and sample preparation

Purified FGFR1 KD variants were incubated with Tet1-TAMRA-X (50 μM, 20 equiv.) or FlAsH-EDT_2_ (100 μM, 40 equiv.) at 25 °C for 1 hour and labelling confirmed by in-gel fluorescence. For smFRET experiments, the labeled proteins were incubated in the presence of ATP or ADP (150 μM) or in the absence of nucleotides at 25 °C for 1 hour, prior to immobilization. Further detail of protein labeling and sample preparation is described in Supplementary Material.

### Single molecule FRET (smFRET) measurements

SmFRET measurements were performed with a custom-built objective type laser-illuminated total internal reflection fluorescence microscope with an Olympus 1.49 N.A. 100X oil immersion objective. The proximity ratio, *E*_PR_, corrected for background signal, crosstalk (*L*), and direct excitation (*D*) was calculated using the fluorescent intensities from video frames according to the following equation: 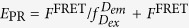 where 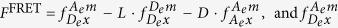 is the signal in the acceptor detection channel after donor excitation, 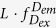 is the *L* corrected signal in the donor channel after donor excitation and 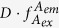 is the signal in the acceptor detection channel (negligible donor excitation). Further information about the TIRF microscope, smFRET procedures and TR-FRET can be found in Supplementary Material.

### Computational calculations

Enhanced-sampling molecular dynamic simulations (parallel-tempering metadynamics, PTMetaD) were according to Bussi *et al*.^43^ and are described further in Supplementary Material.

## Additional Information

**How to cite this article**: Perdios, L. *et al*. Conformational transition of FGFR kinase activation revealed by site-specific unnatural amino acid reporter and single molecule FRET. *Sci. Rep.* **7**, 39841; doi: 10.1038/srep39841 (2017).

**Publisher's note:** Springer Nature remains neutral with regard to jurisdictional claims in published maps and institutional affiliations.

## Supplementary Material

Supplementary Information
