# Supplementary material for: Conformational transition of FGFR kinase activation revealed by site-specific unnatural amino acid reporter and single molecule FRET
Source: Sci Rep. 2017 Jan 3;7:39841. doi: 10.1038/srep39841 (PMC5206623; doi:10.1038/srep39841)
Supplement: Supplementary Information [file srep39841-s1.pdf]

Conformational transition of FGFR kinase activation revealed by site-specific unnatural amino acid reporter and single molecule FRET

Louis Perdios, Alan R. Lowe, Giorgio Saladino, Tom D. Bunney, Nethaji Thiyagarajan, Yuriy Alexandrov , Christopher Dunsby, Paul M. W. French, Jason W. Chin, Francesco L. Gervasio, Edward W. Tate and Matilda Katan1

Supplementary Material

Supplementary Materials and Methods

Supplementary References

Supplementary Figure Legends

Supplementary Figures S1-S6

Supplementary Materials and Methods

Cloning, expression and purification of recombinant proteins

Construction of vectors

To generate the kinase expression vector, an epitope tagged FGFR1 insert, used in our previous studies [1] was restriction digested and then ligated into pCDF PylT plasmid [2]. A tetracysteine (TC) sequence was introduced between the FGFR1 kinase domain and the COOH-terminal His tag using standard PCR splicing techniques. All site directed mutagenesis was performed using the Quickchange mutagenesis kit (Stratagene), following manufacturers protocols. Specifically, the amber stop (TAG) codons, H589TAG and L662TAG were introduced to allow the incorporation of artificial amino acids at these positions. All constructs were fully sequenced to ensure PCR procedures had not incorporated unwanted changes. The final expression plasmids (fully described as *p*2xStrpII.*SUMO*.FGFR1KD3YTAG*.TC.His_{10}.*PylT* plasmids) abbreviated as pDuoGlo-$FGFR1^{H589TAG}$ and pDuoGlo-$FGFR1^{L662TAG}$ were then used for protein expression.

Expression of FGFR1KD3Y variants with incorporated unnatural amino acid BCN-Lysine (BCNK)

E. coli strain BL21 (DE3) was transformed with *pBKBCNRS* (which encodes *Mb*BCNRS) [3] and the FGFR1 expression clones pDuoGlo-$FGFR1^{H589TAG}$ or pDuoGlo-FGFR1 ^{L662TAG}. Transformed cells were selected overnight on agar plates comprising terrific broth (TB) containing carbenicillin (100 µg/mL) and spectinomycin (75 µg/mL). Subsequently, individual colonies were used to inoculate 2 mL of TB with antibiotics and then the following day, this overnight culture was used to inoculate 200 mL of TB supplemented with carbenicillin (50 µg/mL) and spectinomycin (35 µg/mL) and incubated at 37 ^{o}C with constant shaking. When the cell density (OD_{600}) reached 0.4 to 0.5, a solution of BCN-Lysine (BCNK) in H_2O (with equimolar NaOH) was added to the culture to a final concentration of 2 mM. At an OD_{600} of 1.0 to 1.2, cultures were cooled to 15 °C for one hour and then expression was induced with 400 µM IPTG for approximately 16 hours. Cells were harvested by centrifugation and frozen at -80 ^{o}C until processed.

Purification of recombinant proteins

Pellets derived from 200 mL cultures were thawed on ice and resuspended in 8 mL of chilled lysis buffer (25 mM Tris-HCl, 250 mM NaCl, 40 mM imidazole, 100 µg/ml lysozyme, pH 8.0) and supplemented with EDTA-free protease inhibitor cocktail (Roche). Once resuspended, 2 mL of 10% (v/v) Triton-X-100 and 1 Ku of bovine pancreatic DNAse I was added and the lysate was placed on the orbital shaker at 150 rpm at 4 °C for 1 hour. Cell lysates were clarified by centrifugation at 13,000 rpm at 4 °C for 1 hour in an SS34 rotor (Sorvall). The clarified lysate was loaded onto a 1-mL HisTrap FF (GE Healthcare) on an Akta Explorer system (GE Healthcare) using His Buffer A (25 mM Tris-HCl, 500 mM NaCl, 40 mM Imidazole, 1 mM TCEP, pH 8.0). His-tagged proteins were separated by applying a linear imidazole gradient [from His Buffer A to His Buffer B (25 mM Tris-HCl, 500 mM NaCl, 500 mM imidazole, 1 mM TCEP, pH 8.0)] over 5 column volumes. The post-HisTrap eluent was loaded onto a 1 mL StrepTactin Sepharose High Performance (StrepTrap HP, GE Healthcare) column on an Akta Explorer system using Binding Buffer A (50 mM Tris-HCl, 150 mM NaCl, pH 8.0). StrepII-tagged proteins were collected by eluting with step gradient from Binding Buffer A to Elution Buffer B (50 mM Tris-HCl, 150 mM NaCl, 5mM desthiobiotin, pH 8.0) over 8 column volumes. Protein concentrations from the eluent were measured with a Nanodrop (ThermoScientific). Fractions containing the target protein were pooled, concentrated in spin concentrators (Vivaproducts) and then further purified by size exclusion chromatography (SEC) on a Superdex 200 10/300 GL column (GE Healthcare) using Gel Filtration Buffer (25 mM Tris-HCl, 150 mM NaCl, 1 mM TCEP, pH 8.0). Fractions were collected, combined and concentrated in spin concentrators, snap frozen in liquid nitrogen and stored at -80 °C. Purified proteins were analyzed by 10% SDS-PAGE.

Kinase assays

In vitro kinase assays of FGFR1 synthetic variant proteins were carried out using the ADP-Glo™ Kinase Assay (Promega) as described previously [4]. The assays were carried out at 25 °C in 40 mM Tris-HCl pH 8.0, 20 mM NaCl, 20 mM $MgCl_2$, 1 mM $MnCl_2$, 1 mM TCEP, and 100 µM Na_3VO_4, in a total volume of 60 µL (15 µL kinase reaction; 15 µL ADP-Glo™ Reagent; 30 µL Kinase Detection Reagent) in solid white, flat-bottom 96-well plates. In some assays, Poly (E_4Y_1) peptide (Sigma-

Aldrich) was used as kinase substrate. Kinase reactions were stopped after 90 min of incubation for autophosphorylation reactions and 45 min of incubation for time-course with substrate. ATP-to-ADP standard conversion curves, Z-values and signal-to-background ratio were calculated according to the manufacturer's published procedure. For determinations of the K_m for ATP ($K_{m,ATP}$), reactions contained a peptide concentration of 0.5 mg/mL and ATP at varying concentrations from 10 to 240 µM. Data was then fitted using non-linear regression to the Michaelis–Menten equation. The optimal kinase amount was determined when 50% of substrate conversion was achieved (half maximal reaction velocity, ½V_{max}), and assays were carried out at the apparent $K_{m,ATP}$, at 150 µM ATP. Negative control experiments were performed in the absence of ATP, substrate, enzyme and positive controls were performed with FGFR1 WT (data not shown). Luminescence output was recorded using BMG Labtech FLUOstar Optima plate reader at 520nm. Curve fitting for was performed using GraphPad Prism® software.

SDS-PAGE and Western blotting

Proteins were separated using standard procedures and transferred onto polyvinylidene fluoride (PVDF) membrane for 16 hours at 30 V at 4 °C. For immunoblotting, the membranes were blocked with 5% non-fat dry milk (Sigma) in TBS pH 7.5 containing 0.1% Tween-20 (Sigma-Aldrich) (TBS-T) for 30 – 60 min with agitation. Membranes were incubated with the indicated primary antibodies for 1 hour at 25 °C or overnight incubation at 4 °C. Membranes were washed three times for 5 min in TBS-T followed by incubation with the required conjugate diluted in 5% milk/TBS-T for 1 hour with agitation. Blots were subject to three washes in TBS-T and developed using with ECL prime detection kit (Pierce) and exposed to Hyperfilm ECL (Amersham Biosciences). Protein transfer to the membrane was then visualised by Amido Black staining.

In vitro labeling of purified proteins with Tet1-TAMRA-X and/or FlAsH-EDT$_2$

Purified recombinant proteins (~2.5 µM in 25 mM Tris-HCl, 150 mM NaCl, 1 mM TCEP, pH 8.0) were incubated with Tet1-TAMRA-X (50 µM, 20 equiv.) and/or FlAsH-EDT$_2$ (100 µM, 40 equiv.) at 25 ^{o}C for 1 hour and analyzed by SDS-PAGE. The SDS-PAGE gels were scanned with a Typhoon FLA 9500

biomolecular imager (GE Life Sciences) to visualize in-gel fluorescence using the Cy2 settings (λex: 473 nm, λem: 530 nm) for FlAsH-EDT_2 and the Cy3 setting (λex: 532 nm, λem: 570 nm) for Tet1-TAMRA-X and then stained with Coomassie Blue. For time course in-vitro labeling reactions, ~50 pmol of purified protein (~2.5 μM in 15 μL of 25 mM Tris-HCl, 150 mM NaCl, 1 mM TCEP, pH 8.0) was incubated with 1.5 nmol of Tet1-TAMRA-X (100 μM, ≥30 fold excess) or 1.5 nmol of FlAsH-EDT_2 (100 μM, ≥30 fold excess). Samples were incubated at 25 ^{o}C for 15 min to 24 hours and the reaction was terminated by mixing with SDS-PAGE sample buffer and heating for 5 min to 95 ^{o}C before analyzing by 10% SDS-PAGE. The amounts of labeled proteinswere quantified by scanning with a Typhoon imager and using ImageJ to calculate the on-protein labeling rate constant under pseudo-first order kinetics.

Single molecule FRET (smFRET) measurement

Imaging instrumentation

All single-molecule imaging was conducted using a custom-built microscope, based on a fully motorised Olympus IX81 base. Four lasers (100 mW 405 nm Coherent Obis, 100 mW 488 nm Coherent Sapphire, 150 mW 561 nm Coherent Sapphire and a 150 mW Toptica iBeam Smart), each with their own shutter control, were expanded to the same diameter and combined using a series of dichroic mirrors into a single free-space beam. Half-wave plates were used to adjust the polarisation before passing the beams through an Acousto-Optical Tunable Filter (AA Optoelectronics) to quickly modulate laser power. The combined beams were again expanded and launched into a single-mode optical fibre (Thorlabs PM-S405-XP) using an Olympus (0.1 N.A. 10x Air) objective lens. The output of the optical fibre was collimated using an achromatic parabolic mirror collimator (Thorlabs) and passed through a quarter-wave plate to circularly polarise the beam. The free beam then passed through the "TIRF" lens (L1, Thorlabs 200 mm achromatic doublet) and was focused directly onto the back focal plane of the objective lens. This entire subsystem was mounted on a micrometer translation stage to adjust the TIRF angle. The beam was reflected to the objective lens via a multi- edge dichroic filter (Semrock Di01-R405/488/561/635-25x36). The objective lens was an oil-immersion Olympus TIRF apochromatic objective lens (1.49 N.A. 100x Oil). Actively cooled electron-multiplying charged

coupled device (EMCCD) cameras (Andor iXon Ultra) were coupled to the camera port of the microscope via an additional 1.5x magnifying relay. An additional dichroic mirror (Semrock FF560-FDi01-25x36) in the 4f relay was used to simultaneously image the second colour. Bandpass filters (BP1: Semrock FF01-520/35-25 and BP2: Semrock FF01-617/73-25) in front of the two cameras selected the appropriate dye emission. Typically, camera acquisition was at 20 Hz. Laser shutter, acousto-optical tunable filter (AOTF) and camera firing were synchronised using a Data Translation DT9834 data acquisition (DAQ) module, using the internal clock to provide synchronised TTL pulses. Sample positioning was controlled via a motorised micrometer stage (Physik Instrumente) with a XYZ-Nanopositioning stage (Physik Instrumente). All software for microscope control was written in C++ and Python.

Sample and oxygen scavenging buffer preparation

Purified His-tag proteins (0.5 µM) in kinase reaction buffer, KRB (40 mM Tris-HCl, 20 mM NaCl, 20 mM $MgCl_2$, 1 mM $MnCl_2$, 1 mM TCEP, pH 8.0) were labeled with Tet1-TAMRA-X (50 µM) and/or FlAsH-EDT_2 (100 µM) and incubated in the presence or absence of ATP (150 µM, UltraPure ATP, Promega) at 25°C for 1 hour. Non-phosphorylated samples were supplemented with lambda protein phosphatase (λPPase) to release any phosphate groups from phosphorylated serine, threonine and tyrosine residues and phosphorylated samples with Na_3VO_4 (100 µM) to preserve the protein phosphorylation state. Chelated copper (Cu^{2+}) surface glass coverslips with low density PEG coating (Microsurfaces) were mounted to bottomless channel slides with a self-adhesive underside (sticky-Slide VI$^{0.4}$, IBIDI) and the flow chambers assembled were used to perfuse and immobilise purified His-tag proteins.

Labeled samples were diluted to a suitably low concentration (typically 50 – 75 pM) to distinguish one molecule from another, avoid aggregate formation on the slide and to capture 200 – 400 immobilised molecules per field of view. 70 µL of the picomolar dilution was introduced to the flow chamber and incubated in the dark at 25°C for 30 min. The flow chamber was washed with KRB (3x70 µL) and the solution was exchanged with imaging buffer (70 µL), an enzymatic oxygen scavenging system (0.8%

w/v D-glucose, 1 mg/mL (165 U/mL) glucose oxidase, and 0.04 mg/mL (2170 U/mL) catalase, all Sigma-Aldrich) with a triplet state quencher, Trolox [(±)-6-Hydroxy-2,5,7,8-tetramethylchromane-2-carboxylic acid] (2mM, Sigma-Aldrich), which enhances the photostability of the dyes [5].

Camera registration

Chromatic aberrations cause a slight misalignment in the images returned from the two cameras. Therefore, we need to register the images to create a mapping from one space to the other. Before every experiment, we imaged immobilised MultiTetraSpec beads (Invitrogen) using both cameras, and used the image data to calculate the correction field using the following method:

1. Crudely align the channels using a normalised cross-correlation
2. Use the non-overlapping regions of the images to discard particles which are not present in both images
3. Fit all of the found PSFs to a Gaussian function to determine their centroids with sub-pixel accuracy in each of the channels
4. Use a Linear Assigned Problem (LAP) solver to find the best mapping between the two sets of particles
5. Calculate a vector field based on their mapping

Once we have registered the channels (Figure S4A), we end up with a set of particles, with positions $\vec{x}_i$ and alignment vectors, $\vec{v}_i$. We can determine the expected position in the second channel for any new particle ($\vec{x}_{new}$) using the weighted (based on distance) sum of the vectors in the vector field:

$$\vec{x}^b_{new} = \vec{x}^a_{new} + \vec{v}_w$$

Where $\vec{v}_w = \frac{\sum_{i=1}^{n} \vec{v}_i / d_i}{\sum_{i=1}^{n} d_i}$ and $d_i = \|\vec{x}_i - \vec{x}^a_{new}\|$.

Data acquisition

We utilised an Alternating-Laser EXcitation (ALEX) illumination scheme [6], coupled with objective type TIRF microscopy to image the individual proteins, and verify their dye labeling stoichiometry. Utilising the external D/A converter, an alternating sequence of laser pulses (12.5 mW 488 nm laser, 50 mW for the 561 nm laser) was generated, and synchronized with the both camera's exposure (typically 50 ms, at 20Hz) using TTL pulses. The cameras were operated at a "real-gain" of 750, and cooled to a temperature of -80 ^{o}C. Movies were recorded using the camera's full-frame (512x512 pixels) and streamed for up to 90 s.

Data analysis

Data analysis was performed according to Hohlbein et al. [7]. Here we briefly describe the methods. The process proceeds as follows:

1. Acquire ALEX data and separate into Donor excitation and Acceptor excitation
2. Align the two image sets to account for chromatic aberration
3. Find candidate molecules in the direct excitation of Acceptor channel
4. Find their centroids and extract a window around the molecules
5. Correct for background fluorescence
6. Correct for dye cross-talk
7. Calculate the proximity ratio FRET (E_{PR}) and stoichiometry (S)

Utilising alternating laser excitation, we generate a movie containing an alternating sequence of frames, such that in the odd frames we illuminate the sample with the 488 nm laser (Donor excitation, D_{ex}) and gather information about $f_{D_{ex}}^{D_{em}}$ and $f_{D_{ex}}^{A_{em}}$ and in the even frames we illuminate the sample with the 561 nm laser (Acceptor excitation, A_{ex}) and gather information about $f_{A_{ex}}^{A_{em}}$. These movies are then aligned to account for chromatic aberrations.

We initially select molecules for further analysis, by localising bright spots (corresponding to the Point Spread Function, PSF, of a single molecule) found in the $f_{A_{ex}}^{A_{em}}$ channel, which possess brightness above a threshold value and corresponding to molecules containing a single Tet1-TAMRA-X dye.

Next we calculate the centroid of the molecules by fitting the PSF to a symmetrical two-dimensional Gaussian function. For a sub-wavelength diameter fluorescent molecule, fitting of the PSF to a Gaussian function yields the highest accuracy and precision of localisation, thus:

$$f(x,y) \approx Ae^{-\left(\frac{(x-x_0)^2}{2\sigma_x^2}+\frac{(y-y_0)^2}{2\sigma_y^2}\right)} + B$$

where *A* is the amplitude, *B* is the background, x_0 and y_0 are the mean *x* and *y* positions, and σ_x and σ_y are the standard deviations in *x* and *y* (where *x* = *y* for symmetrical Gaussian functions). We then extract a stack of windows (sufficient in size to contain the entire PSF, ±3 σ_{xy} around this centroid, through each frame of the movie, in both D_{ex} and A_{ex} channels.

Next we calculate the integrated intensity (I_{em}), and background signal (I_{bg}) in each frame in the stack of windows, and use this to correct for local background fluorescence and CCD noise. The background corrected values of fluorescence are calculated by subtracting the background signal, such that $f = I_{em} - I_{bg}$. Now, we have three background corrected trajectories for each identified molecule, $f_{D_{ex}}^{D_{em}}$, $f_{D_{ex}}^{A_{em}}$ and $f_{A_{ex}}^{A_{em}}$.

Finally, we correct for "dye-crosstalk". In this experiment, there are two types of dye-crosstalk correction factor that we need to take in to account, (i) Leakage of donor emission captured in the acceptor channel, and (ii) Direct excitation of the acceptor by the donor excitation laser. To calculate the correction factor for leakage (L), we measure the fluorescence signal in the donor and acceptor channels using protein labeled with only the donor fluorophore. We can then calculate correction factor *L* as:

$$L = E_D/(1 - E_D)$$

$$\text{Where } E_D = \frac{f_{D_{ex}}^{A_{em}}}{f_{D_{ex}}^{D_{em}} + f_{D_{ex}}^{A_{em}}}$$

Yielding a correction factor *L*, for FlAsH-EDT$_2$, of: 0.19.

Next we need to calculate the correction factor for direct excitation of the acceptor fluorophore using the donor excitation wavelength (*D*). We measure the stoichiometry for protein labeled with only the acceptor fluorophore, S_A, calculating the correction factor *D* as:

$$D = S_A/(1 - S_A)$$

$$\text{Where } S_A = \frac{f_{D_{ex}}^{D_{em}} + f_{D_{ex}}^{A_{em}}}{f_{D_{ex}}^{D_{em}} + f_{D_{ex}}^{A_{em}} + f_{A_{ex}}^{A_{em}}}$$

Yielding a correction factor *D*, for Tet1-TAMRA-X, of: 0.18.

Using the correction factors, we are now in the position to calculate FRET (*E*) and stoichiometry (*S*). In the paper we report the proximity ratio, E_{PR}, rather than the so-called 'accurate FRET'. This method enables one to observe changes in proximity, but precludes reporting of accurate molecular scale distance changes. E_{PR} is calculated as follows:

$$E_{\text{PR}} = \frac{F^{\text{FRET}}}{f_{D_{ex}}^{D_{em}} + F^{\text{FRET}}}$$

$$\text{Where } F^{\text{FRET}} = f_{D_{ex}}^{A_{em}} - L \cdot f_{D_{ex}}^{D_{em}} - D \cdot f_{A_{ex}}^{A_{em}}$$

The stoichiometry of the complex (*S*) can be calculated as follows:

$$S = \frac{f_{D_{ex}}^{D_{em}} + F^{\text{FRET}}}{f_{D_{ex}}^{D_{em}} + f_{D_{ex}}^{D_{em}} + F^{\text{FRET}}}$$

Time-resolved FRET (TR-FRET)

Fluorescence lifetime decay measurements were made of the purified protein solutions in a quartz cuvette using time-correlated single photon counting (TCSPC) implemented in a multidimensional

spectrofluorometer [8]. The purified proteins (0.5 μM) were tagged with FlAsH-EDT2 (100 μM) or Tet1-TAMRA-X (50 μM) in 40 mM Tris-HCl pH 8.0, 20 mM NaCl, 20 mM MgCl2, 1 mM MnCl2, 1 mM TCEP, and 100 μM Na3VO4 and were incubated in the presence or absence of ATP (150 μM) at 25 oC for 1 hour. Excitation of the FRET pair was performed at 490 nm using the picosecond supercontinuum laser excitation source, and fluorescence emission was detected at 545 nm.

Chemical syntheses

General methods

All solvents and reagents were obtained from commercial suppliers and were used without further purification. BCN-Lysine, (2S)-2-amino-6-(1*R*,8*S*,9r)-Bicyclo[6.1.0]non-4-yn-9-ylmethoxy carbonyl amino hexanoic acid ($C_{17}H_{26}N_2O_4$, 322.4 gmol^{-1}) was synthesised as described previously [2, 9]. Tet1-TAMRA-X was custom synthesised according to [10].

Computational Methods

Molecular dynamics calculations

Molecular dynamics simulations were performed using GROMACS 4.5 [11] with the PLUMED plug-in and the Charmm22* force field [12]. Phosphorylated and non-phosphorylated FGFR systems were solvated with TIP3P water molecules in a dodecahedral box. Both systems were minimized with 10000 steps conjugated gradient and equilibrated for 10 ns in the NPT ensemble. Production runs were carried out in the NVT ensemble using the velocity-rescale thermostat [13] with a timestep of 2 fs. The PME algorithm was used for electrostatic interactions with a cut-off of 1.2 nm while a single cut-off of 1.2 was used for Van der Waals interactions.

Enhanced Sampling

Parallel Tempering Metadynamics (PT-metaD) [14] was performed for FGFR systems using 20 replicas in the 298.0 K - 337.5 K temperature range. The well-tempered metadynamics algorithm was used, so that gaussian height is progressively decreased throughout the run [15]. The initial gaussian height was set at 10 kJ/mol with a bias factor of 10. The two collective variables used were the

distance in contact map space to the inactive A-loop conformation and the distance in contact map space to the active conformation, as in previous simulations [4, 16], with a sigma of 0.5 for both CVs. Note that the CVs are adimensional. A total of 450 ns was needed to fully converge the free energy surfaces of the two systems.

Supplementary References

1. Bunney, T.D., et al., *Structural and functional integration of the PLCgamma interaction domains critical for regulatory mechanisms and signaling deregulation.* Structure, 2012. **20**(12): p. 2062-75.
2. Lang, K., et al., *Genetic Encoding of bicyclononynes and trans-cyclooctenes for site-specific protein labeling in vitro and in live mammalian cells via rapid fluorogenic Diels-Alder reactions.* J Am Chem Soc, 2012. **134**(25): p. 10317-20.
3. Lang, K., L. Davis, and J.W. Chin, *Genetic encoding of unnatural amino acids for labeling proteins.* Methods Mol Biol, 2015. **1266**: p. 217-28.
4. Bunney, T.D., et al., *The Effect of Mutations on Drug Sensitivity and Kinase Activity of Fibroblast Growth Factor Receptors: A Combined Experimental and Theoretical Study.* EBioMedicine, 2015. **2**(3): p. 194-204.
5. Krishnamurthy, N.V., A.R. Reddy, and B. Bhudevi, *Wavelength dependant quenching of 2,5-diphenyloxazole fluorescence by nucleotides.* J Fluoresc, 2008. **18**(1): p. 29-34.
6. Kapanidis, A.N., et al., *Fluorescence-aided molecule sorting: analysis of structure and interactions by alternating-laser excitation of single molecules.* Proc Natl Acad Sci U S A, 2004. **101**(24): p. 8936-41.
7. Hohlbein, J., T.D. Craggs, and T. Cordes, *Alternating-laser excitation: single-molecule FRET and beyond.* Chem Soc Rev, 2014. **43**(4): p. 1156-71.
8. Manning, H.B., et al., *A compact, multidimensional spectrofluorometer exploiting supercontinuum generation.* J Biophotonics, 2008. **1**(6): p. 494-505.

9. Dommerholt, J., et al., *Readily accessible bicyclononynes for bioorthogonal labeling and three-dimensional imaging of living cells.* Angew Chem Int Ed Engl, 2010. **49**(49): p. 9422-5.

10. Lang, K., et al., *Genetically encoded norbornene directs site-specific cellular protein labelling via a rapid bioorthogonal reaction.* Nat Chem, 2012. **4**(4): p. 298-304.

11. Pronk, S., et al., *GROMACS 4.5: a high-throughput and highly parallel open source molecular simulation toolkit.* Bioinformatics, 2013. **29**(7): p. 845-54.

12. Piana, S., K. Lindorff-Larsen, and D.E. Shaw, *How robust are protein folding simulations with respect to force field parameterization?* Biophys J, 2011. **100**(9): p. L47-9.

13. Bussi, G., D. Donadio, and M. Parrinello, *Canonical sampling through velocity rescaling.* J Chem Phys, 2007. **126**(1): p. 014101.

14. Bussi, G., et al., *Free-energy landscape for beta hairpin folding from combined parallel tempering and metadynamics.* J Am Chem Soc, 2006. **128**(41): p. 13435-41.

15. Barducci, A., G. Bussi, and M. Parrinello, *Well-tempered metadynamics: a smoothly converging and tunable free-energy method.* Phys Rev Lett, 2008. **100**(2): p. 020603.

16. Marino, K.A., L. Sutto, and F.L. Gervasio, *The effect of a widespread cancer-causing mutation on the inactive to active dynamics of the B-Raf kinase.* J Am Chem Soc, 2015. **137**(16): p. 5280-3.

Supplementary Figure Legends

Figure S1. External fluorogenic reagents and their corresponding labeling residues

(a) Chemical reaction scheme of tetracysteine sequence and FlAsH-EDT_2. The backbone structure of the optimised tetracysteine sequence (FLNCCPGCCMEP) bound to a derivative of FlAsH is defined by a hairpin-like turn.

(b) Structural formulae of the bicyclononyne (BCN)-containing unnatural amino acid, BCN-Lysine, (2S)-2-amino-6-(1*R*,8*S*,9r)-Bicyclo[6.1.0]non-4-yn-9-ylmethoxy carbonyl amino hexanoic acid

($C_{17}H_{26}N_2O_4$, 322.4 $gmol^{-1}$), and tetrazine-tetramethylrhodamine fluorophore 9 (5-TAMRA-X), ($C_{45}H_{43}N_{11}O_6$, 834.5 $gmol^{-1}$).

Figure S2. Labeling with FlAsH-EDT_2 and Tet1-TAMRA-X

(a) Time course of labeling to calculate on-protein labeling rate constants. Each measurement was carried out twice and the error bars represent the standard deviation (SD). SDS-PAGE fluorescent images were visualised on a Typhoon Imager and Coomassie stained loading controls are shown.

(b) Identification of pY residues. Phosphopeptide analysis (LTQ Velos mass spectrometer) was performed following in vitro phosphorylation. Peptides are derived from trypsin based workflows.

Figure S3. Detailed schematic of single-molecule FRET microscope layout

An Olympus IX81 base with high NA objective lens is used as the body of the microscope. Laser illumination is coupled into the system using a single-mode polarisation maintaining fibre. Two EMCCD cameras are attached to the camera port via a relay, enabling simultaneous two-colour imaging. Cameras and lasers are synchronised using an external signal generator. An infrared laser autofocus system is coupled via the camera port to maintain a focus lock during image acquisition.

Figure S4. Camera registration and examples of single-molecule fields of view and FRET

(a) An example overlay of the two cameras showing polychromatic beads adsorbed onto the coverglass. (I) Chromatic aberrations cause a relative shift in the positions of the beads. (II) The vector field describing the chromatic aberrations can be determined by mapping the correspondences between the two cameras, in order to calculate the offset at any given position.

(b) An example of FlAsH-labeled proteins (left) and of TAMRA-X-labeled proteins (right)

(c) An example of smFRET

Figure S5. Comaparison of H589BCNK.TC and L662BCNK.TC proteins

(a) Superimposed $E_{PR}S$ 2D histogram/contour plots of non-phosphorylated (N, blue) and phosphorylated (P, orange) samples for the two FGFR KD varians.

(b) Model for relative distances of fluorophores in an inactive and active FGFR1 KD

A model of FGFR1 KD incorporates elements (BCNK and TC-motif) used for labeling with

FlAsH (green) and TAMRA-X (red and orange). Relative distances between FlAsH and TAMRA-X in H589BCNK.TC and L662BCNK.TC proteins (orange and red lines, respectively) are indicated in inactive (left) and active (right) structures of FGFR1 KD. The key below the structures describes the relative distance between fluorogenic centres BCNK/Tet1-TAMRA-X and FlAsH/TC. A comparison can be drawn between the non-phosphorylated and phosphorylated distances of the fluorophores in L662BCNK.TC and, similarly, of H589BCNK.TC. This model is consistent with the data shown in (a) including a larger difference between inactive and active state for the L662BCNK.TC protein.

Figure S6. Purification and characterization of H589BCNK.TC and L662BCNK.TC proteins

(a) Purification of H589BCNK.TC and L662BCNK.TC proteins. Samples obtained at different stages of purification were analyzed by SDS-PAGE; lane 1: E.coli pellet before induction, lane 2: E.coli pellet after IPTG induction, lane 3: Clarified lysate, lane 4: Immobilized-Metal Affinity Chromatography (IMAC) Ni2+ eluate, lane 5: Steptavidin Trap affinity purification eluate, lane 6: Size Exclusion Chromatography eluate (top panels). In the main Fig. 2, lanes 4, 5 and 6 contained the same samples as shown here but with equial protein amounts as in lanes 1, 2 and 3.

(b) Following labeling of H589BCNK.TC and SDS-PAGE, the protein was visualized by Coomassie Blue staining.

(c) Samples of H589BCNK.TC and L662BCNK.TC before (- ATP) and after 45 min incubation with ATP (+ ATP) were subjected to SDS-PAGE and Western blotting using anti-pY653/654 antibody (top); the membrane was subsequently stained with Amido Black (bottom). Lane 1: L662BCNK.TC (-ATP); lane 2: L662BCNK.TC (+ATP); lane 3: H589BCNK.TC (-ATP); lane 4: H589BCNK.TC (+ATP).

a

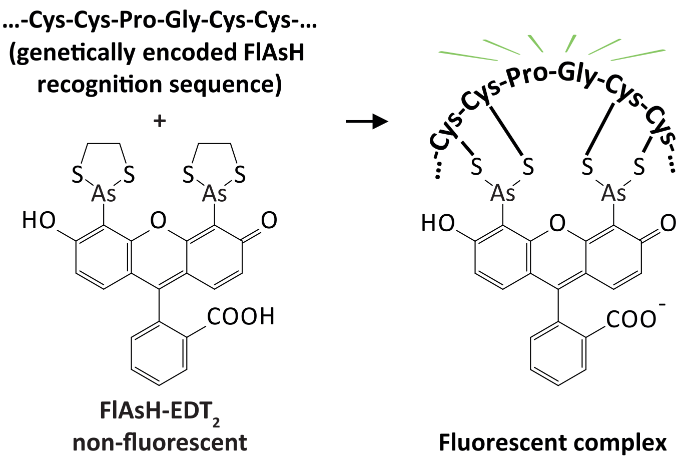

...-Cys-Cys-Pro-Gly-Cys-Cys-...
(genetically encoded FlAsH recognition sequence)
+
FlAsH-EDT$_2$
non-fluorescent
Fluorescent complex

b

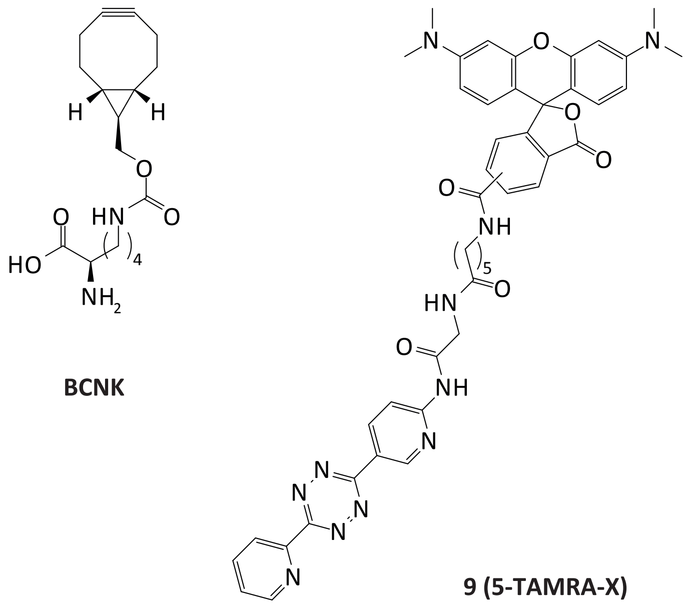

BCNK
9 (5-TAMRA-X)

Figure S1

a

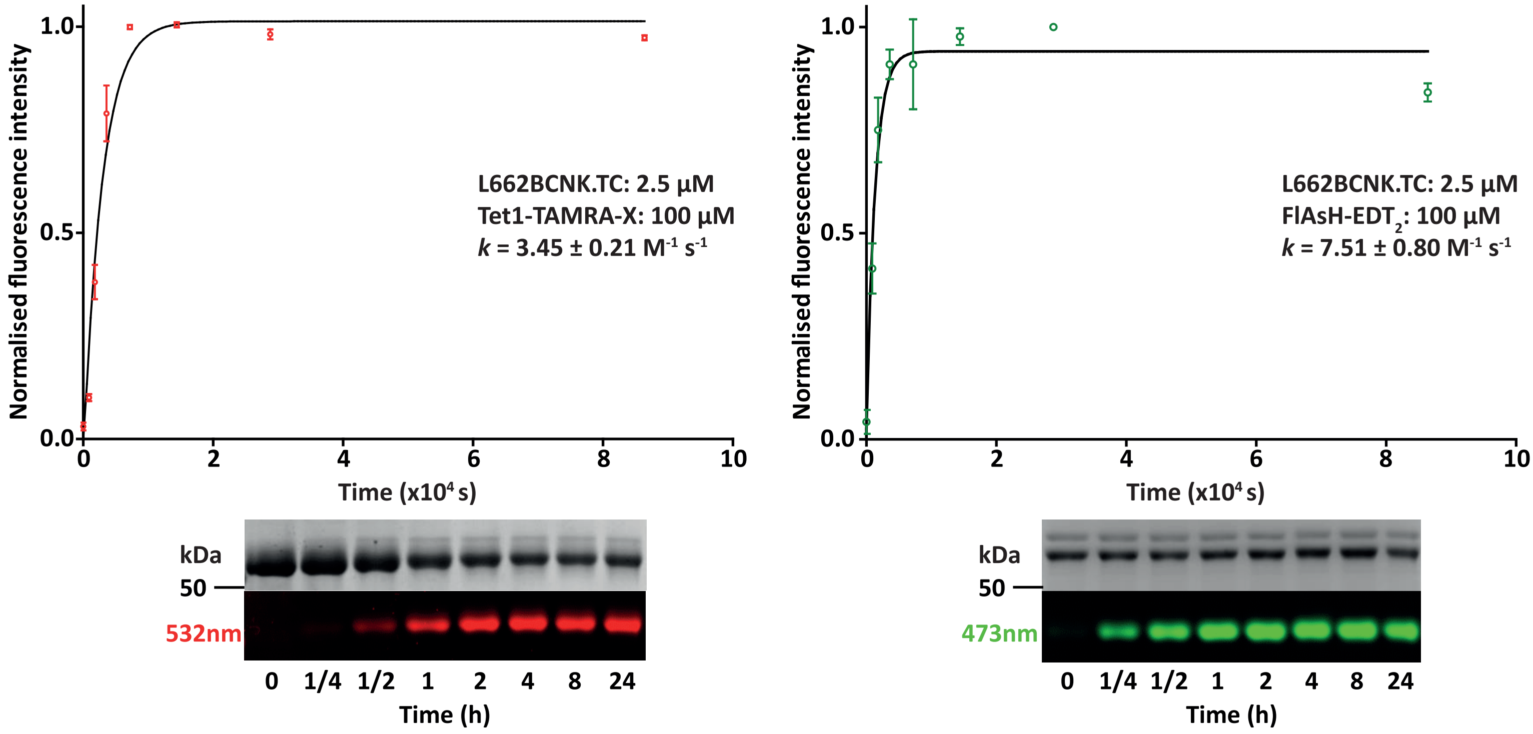

b

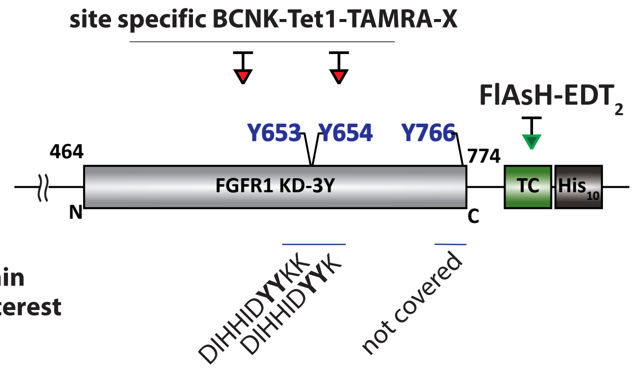

Peptides with Y residues within the highlighted regions of interest

Identification of pY653 and pY654

Sequence	Mass calculated (unmodified)	Mass (2pY)	Confidence (PEP)	Mass error (ppm)	Precursor mass	Charge (z)
DIHHDYYKK	1331. 49	1491.4705	5.52E-09	-0.37317	746.308	2

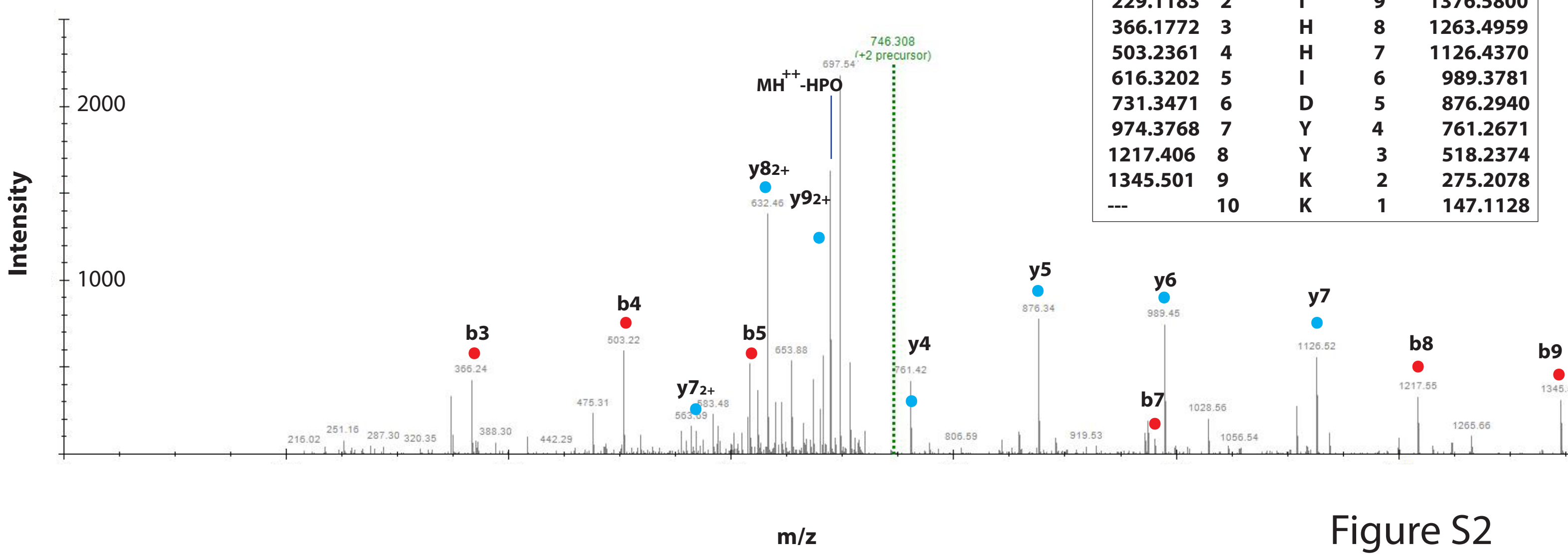

b ions				y ions
---	1	D	10	---
229.1183	2	I	9	1376.5800
366.1772	3	H	8	1263.4959
503.2361	4	H	7	1126.4370
616.3202	5	I	6	989.3781
731.3471	6	D	5	876.2940
974.3768	7	Y	4	761.2671
1217.406	8	Y	3	518.2374
1345.501	9	K	2	275.2078
---	10	K	1	147.1128

Figure S2

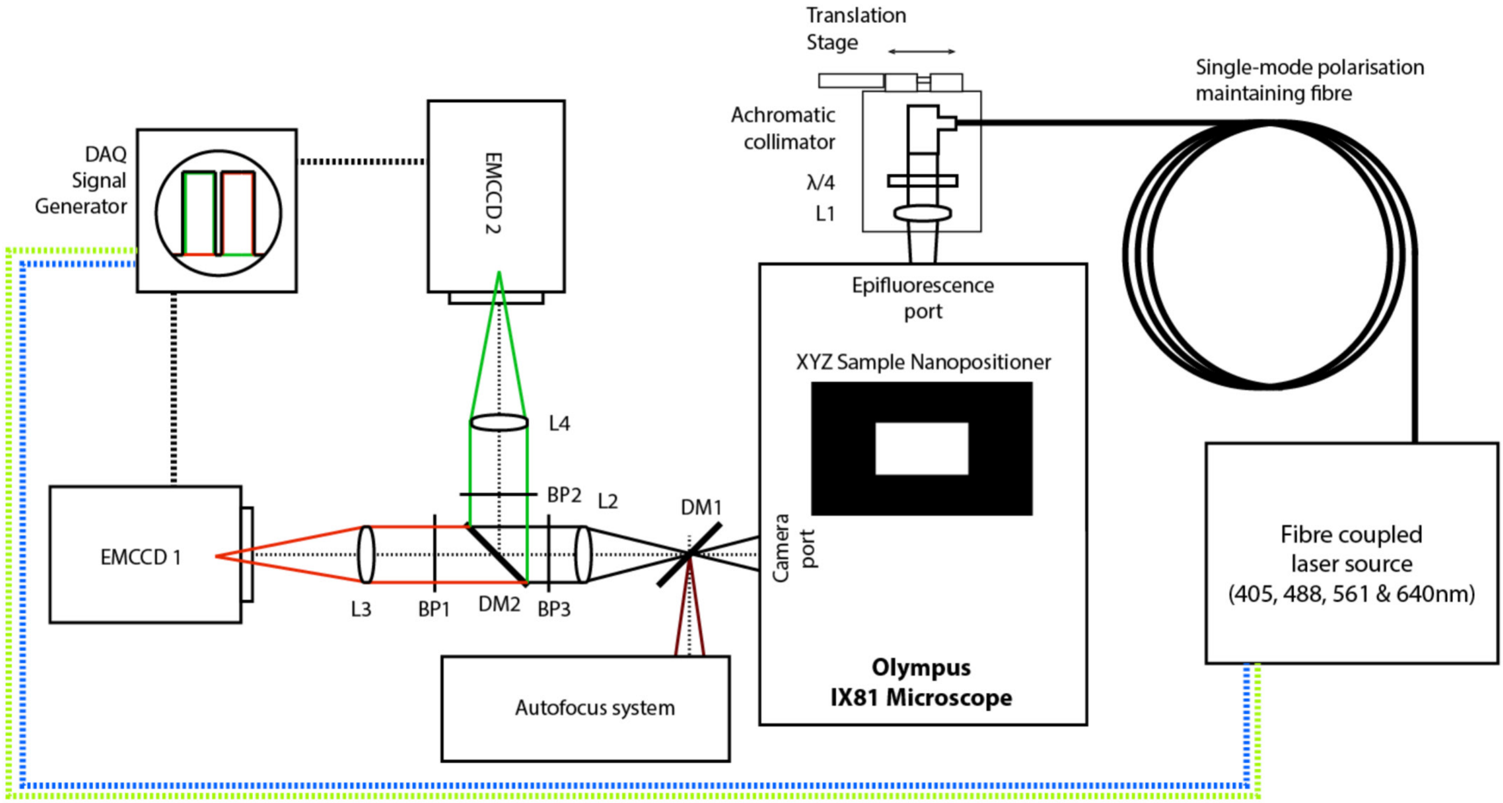
Translation Stage
Single-mode polarisation maintaining fibre
Achromatic collimator
λ/4
L1
DAQ Signal Generator
EMCCD 2
Epifluorescence port
XYZ Sample Nanopositioner
L4
BP2
L2
DM1
Camera port
EMCCD 1
L3
BP1
DM2
BP3
Fibre coupled laser source (405, 488, 561 & 640nm)
Olympus IX81 Microscope
Autofocus system

Figure S3

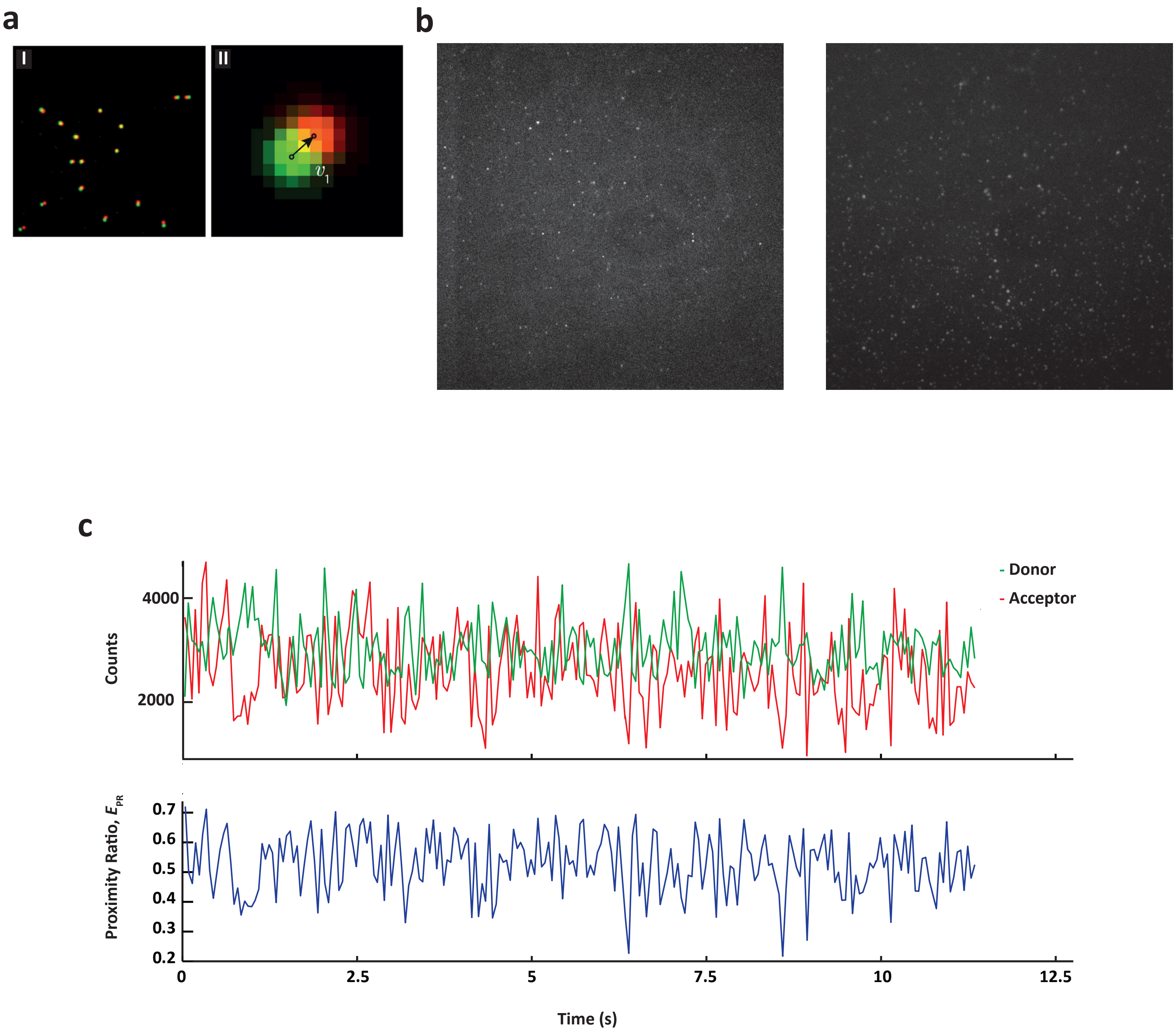
a
I
II
v_1
b
c
Counts
4000
2000
- Donor
- Acceptor
Proximity Ratio, E_{PR}
0.7
0.6
0.5
0.4
0.3
0.2
0
2.5
5
7.5
10
12.5
Time (s)

Figure S4

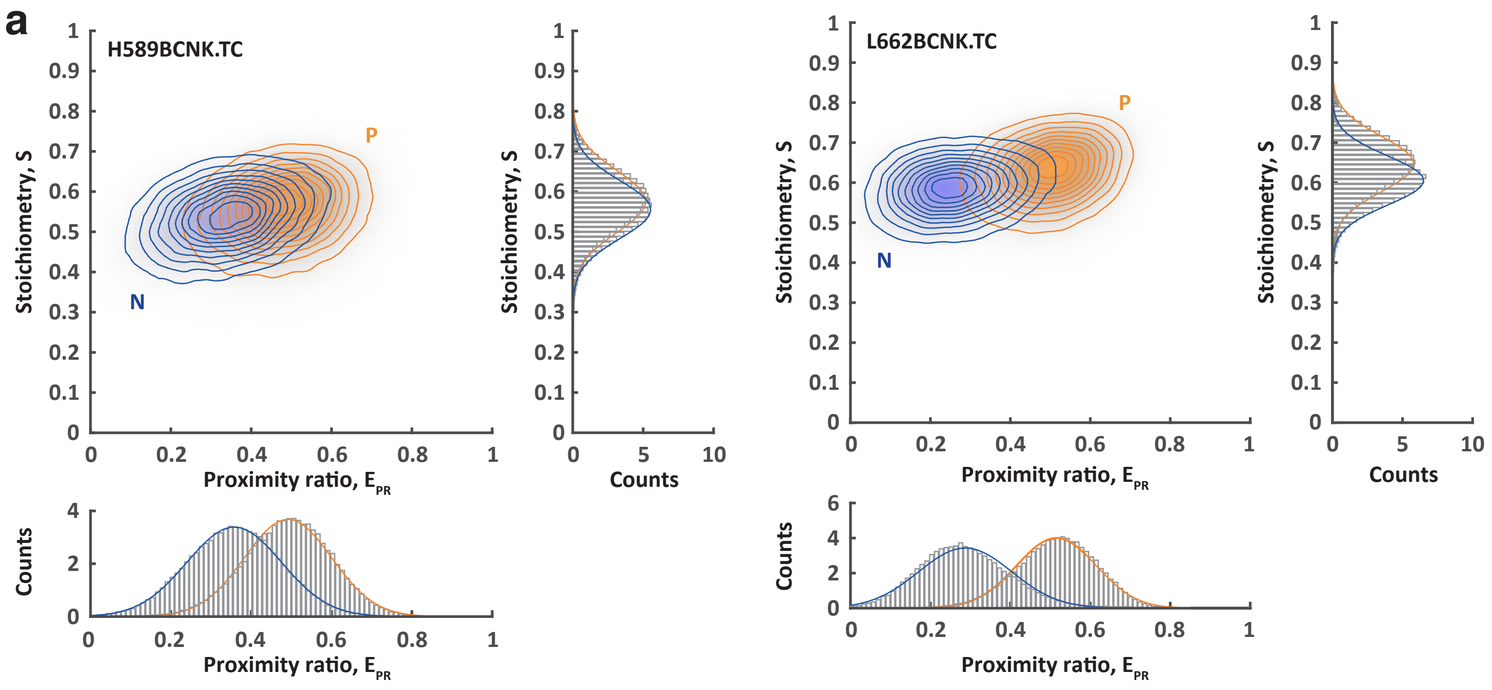
a
H589BCNK.TC
P
N
Stoichiometry, S
Proximity ratio, E_{PR}
Counts
L662BCNK.TC
P
N
Stoichiometry, S
Proximity ratio, E_{PR}
Counts

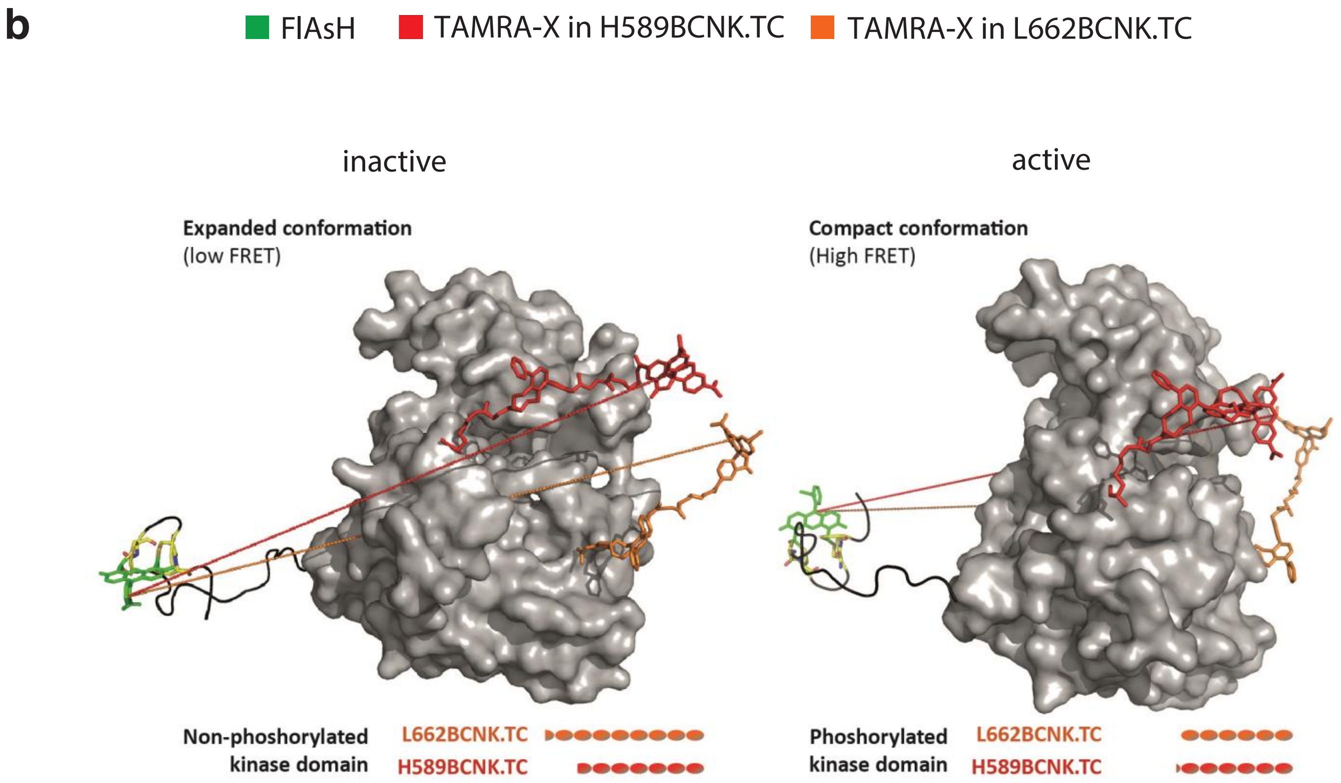
b
FlAsH
TAMRA-X in H589BCNK.TC
TAMRA-X in L662BCNK.TC
inactive
active
Expanded conformation
(low FRET)
Compact conformation
(High FRET)
Non-phoshorylated
kinase domain
L662BCNK.TC
H589BCNK.TC
Phoshorylated
kinase domain
L662BCNK.TC
H589BCNK.TC

Figure S5

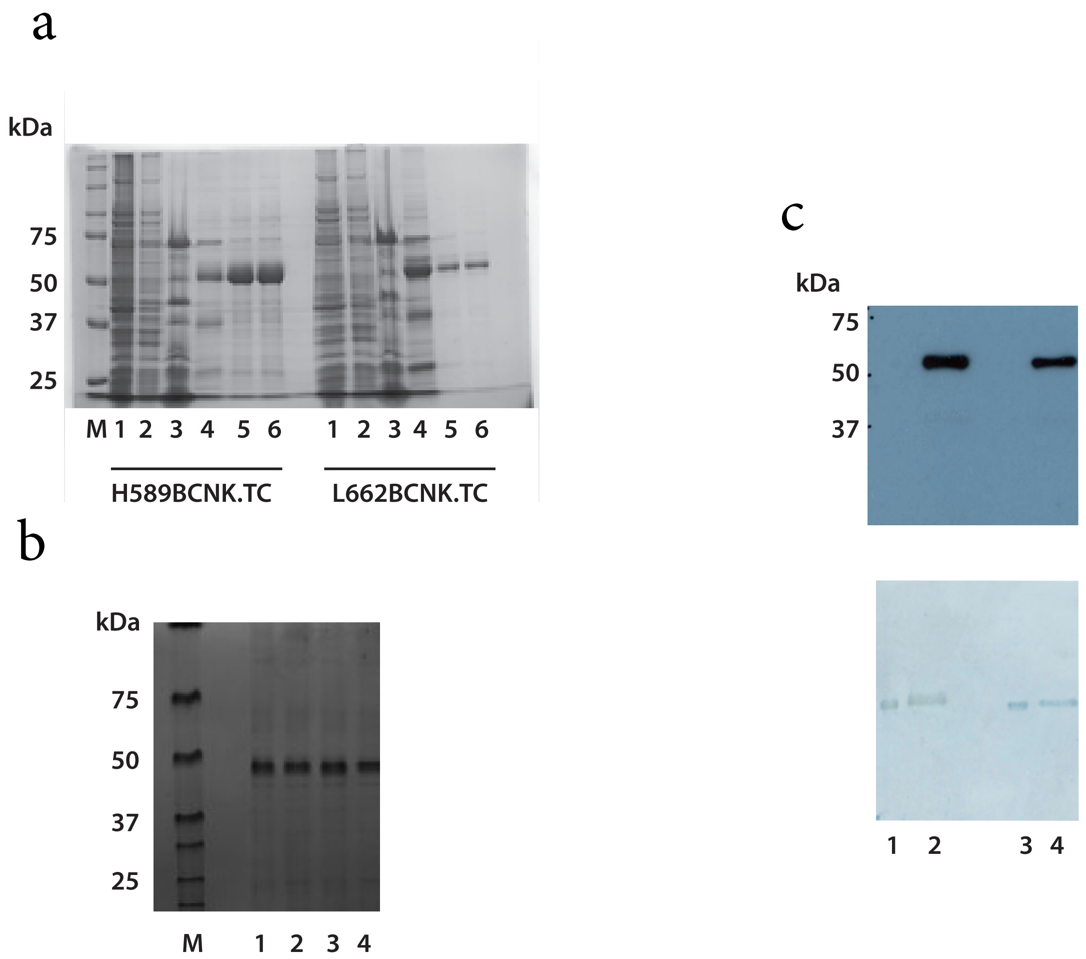
a
kDa
75
50
37
25
M 1 2 3 4 5 6
1 2 3 4 5 6
H589BCNK.TC
L662BCNK.TC
b
kDa
75
50
37
25
M 1 2 3 4
c
kDa
75
50
37
1 2 3 4

Figure S6
